# Supplementary material for: Enabling transparent toxicokinetic modeling for public health risk assessment
Source: PLoS One. 2025 Apr 16;20(4):e0321321. doi: 10.1371/journal.pone.0321321 (PMC12002443; doi:10.1371/journal.pone.0321321)
Supplement: Attachment — Submitted filename: Rebuttal Letter-021025.docx [file pone.0321321.s012.docx]

Rebuttal Letter PONE-D-24-38287

Enabling Transparent Toxicokinetic Modeling for Public Health Risk Assessment

Thank you very much for the thorough feedback on our manuscript and the opportunity to revise. We have undertaken a major revision and significantly reorganized the Introduction, Results, and Methods. We feel that the manuscript is greatly improved. We appreciate any further consideration and feedback that you can offer.

*A rebuttal letter responds to each point raised by the academic editor and reviewer(s).*

# Associate Editor

Thank you for considering our journal for publication of your manuscript. Your manuscript has now been peer-reviewed, could you please take their comments into account and submit a revised version of your manuscript for our consideration?

Please submit your revised manuscript by Feb 06 2025 11:59PM. 1. When submitting your revision, we need you to address these additional requirements.

Please ensure that your manuscript meets PLOS ONE's style requirements, including those for file naming. The PLOS ONE style templates can be found at

https://journals.plos.org/plosone/s/file?id=wjVg/PLOSOne_formatting_sample_main_body.pdf and

https://journals.plos.org/plosone/s/file?id=ba62/PLOSOne_formatting_sample_title_authors_affiliations.pdf

We have revised our manuscript formatting to match these guidelines. In particular we have removed figures from the main document and now provide them as TIFF files.

2. Thank you for stating the following financial disclosure:

[The United States Environmental Protection Agency (EPA) through its Office of Research and Development (ORD) funded the research described here. This project was supported by appointments to the Internship/Research Participation Program at ORD and administered by the Oak Ridge Institute for Science and Education through an interagency agreement between the U.S. Department of Energy and U.S. EPA. J.P.S acknowledges funding support from the U.S. EPA in grant USEPA RD840027].

Please state what role the funders took in the study. If the funders had no role, please state: ""The funders had no role in study design, data collection and analysis, decision to publish, or preparation of the manuscript.""

If this statement is not correct you must amend it as needed.

Please include this amended Role of Funder statement in your cover letter; we will change the online submission form on your behalf."

We have added financial disclosures for non-EPA authors. The financial disclosure provided is the standard preferred by the EPA Office of Research and Development. Several of the researchers are within the EPA ORD (the funder). The new funding statement reads:

*The United States Environmental Protection Agency (EPA) through its Office of Research and Development (ORD) funded the research described here. ORD scientists contributed to the research described here. This project was supported by appointments to the Internship/Research Participation Program at ORD and administered by the Oak Ridge Institute for Science and Education through an interagency agreement between the U.S. Department of Energy and U.S. EPA. J.P.S acknowledges funding support from the U.S. EPA in grant USEPA RD840027*

3. Please expand the acronym “ U.S. EPA” (as indicated in your financial disclosure) so that it states the name of your funders in full.

This information should be included in your cover letter; we will change the online submission form on your behalf.

Done.

4. Thank you for uploading your study's underlying data set. Unfortunately, the repository you have noted in your Data Availability statement does not qualify as an acceptable data repository according to PLOS's standards.

At this time, please upload the minimal data set necessary to replicate your study's findings to a stable, public repository (such as figshare or Dryad) and provide us with the relevant URLs, DOIs, or accession numbers that may be used to access these data. For a list of recommended repositories and additional information on PLOS standards for data deposition, please see https://journals.plos.org/plosone/s/recommended-repositories.

The R package httk is available from Figshare at: https://doi.org/10.23645/epacomptox.6062791.v1

5. Please include captions for your Supporting Information files at the end of your manuscript, and update any in-text citations to match accordingly. Please see our Supporting Information guidelines for more information: http://journals.plos.org/plosone/s/supporting-information.

We have revised our supporting information to include captions at the end of the manuscript.

# Reviewer #1

This manuscript describes the integration and evaluation of new PBTK models into an existing R package (httk) in order to improve in vitro to in vivo extrapolation, which is critical for human health risk assessment. The manuscript is written exceptionally well with clear referals to case studies and model performance evaluations. The manuscript also addresses limitations of the existing package and the improvements that will be implemented by the authors. I do not have any major queries that would impact on the current standard of the manuscript and would therefore suggest acceptance of the manuscript without revision.

Thank you very much for the positive feedback.

# Reviewer #2

This manuscript describes the process of adding new TK models to the httk R package. The topic is relevant, particularly given the increasing reliance on in vitro and in silico methods for chemical risk assessment.

- While the manuscript provides a valuable overview of httk and its functionalities, several major revisions are needed to enhance clarity, accessibility, and impact. Specifically, the manuscript needs to be more concise and focused on the practical aspects of model integration, with less emphasis on background information already available elsewhere.
- A more concrete and worked-through example is essential for readers to grasp the process.

## Major Revisions

- Streamline the Introduction: The introduction is lengthy and contains substantial background on HTTK and risk assessment that could be condensed or removed. Focus on the specific gap this manuscript addresses – the need for clear guidelines on integrating new models into httk – and the benefits of doing so (e.g., community contributions, model standardization, improved reproducibility).

Thank you for the suggestions. We have shortened the introduction and eliminated redundant information wherever possible. Some of the contextual information (that is, the motivation for developing a transparent workflow) has been moved to the discussion.

- Simplify and Refocus the Results Section: The current results section blends model integration details with evaluation results. Separate these clearly. The re-evaluation of the Linakis et al. model is interesting but distracts from the core purpose of the manuscript. Either move this to a supplementary document or significantly shorten it, focusing on specific changes and their impact on RMSLE. Emphasize the practical implications of observed changes for model developers. The discussion of benchmark_httk() is more appropriate for the methods.
- Reorganize the Methods Section: The methods section is currently quite dense and difficult to follow. Reorganize it into more logical subsections with clear headings, following the steps of model integration described in Table 1. Each subsection should describe the specific tasks involved in that step, the relevant httk functions and data, and provide short, illustrative code examples where appropriate. Move the discussion of benchmark_httk() from the Results to the "Model Evaluation" subsection of the Methods.

Potentially divide into 1) Overview Table, 2) Models, Core Fucntionality,

We have jointly reorganized the Methods and the Results sections to address the reviewer’s concerns about these sections. We thank the reviewer for their suggestions. We agree that there was room for improvement. However, while having considered the reviewer’s recommendations, we propose a slightly different reorganization. Our goal is to help readers address everything that would be needed to get their model incorporated into the public release of httk.

The Methods is now organized into four main sections 1) Creating and Integrating a New HTTK Model, 2) Core HTTK Functionality, 3) HTTK Data Library, and 4) Tools for HTTK Evaluation. Section one has been rearranged to closely follow the steps of Table 1 for adding a model. Sections 2-4 of the methods provide descriptions of the methods and data that are available to model developers.

The Results section has been similarly reorganized. There are three sections all using the inhalation PBTK model as an example. The first follows Table 1 and describes how a new model was added. A new Table 2 specifically references the files created and changes made for that model. Section 2 of the Results describe how the performance of that model was characterized – this is a prerequisite for incorporating any model into the public release of httk. The final section of results described how we determine if integration of the model has impacted performance of the rest of the httk package. We believe that this final section is important as part of the Results because developers of new models will be asked to characterize the impact of adding their model.

- Provide a Concrete, Worked-Through Example: The most significant weakness is the lack of a clear, step-by-step example. While the gas inhalation model is mentioned and supplementary files are provided, the manuscript doesn't guide the reader through the process of integration. Include a simplified, illustrative example within the main text, demonstrating the creation of a basic model (even a one-compartment model) and its integration into httk. Show concrete code snippets and explain the purpose of each step. This example should be the central focus of the manuscript.

Thank you for the suggestions. We have added a new table walking the users through the specific steps and files used to implement the inhaltion PBTK model.

- Clarify the Role of MCSim: The manuscript mentions using MCSim for model description but doesn't clearly explain its advantages or provide enough guidance on its use. Expand this discussion and provide a simple MCSim example within the worked-through example mentioned above. If the authors intend to encourage MCSim use for new httk model contributions, this warrants more attention.

The reviewer’s point is well taken. In addition to the new Table 2 (which references the necessary steps including using MCSim), we have added the following discussion of MCSim to the Discussion:

*Sophisticated computer programmers are not necessarily expert biological modelers, and vice versa. There are significant computational advantages to using lower-level model implementations. For example, languages like C or Fortran can be compiled into assembly machine instructions that ran much more rapidly than interpreted languages like R. However, implementing biological models in compilable languages can be tricky. Biological modelers are often more comfortable with descriptive modeling languages which match human expectations (such as ACSL or Berkeley Madonna) rather than languages better suited for rapid computation [38]. Therefore, it is of great use that open source tools like MCsim [39] provide the ability to translate a descriptive language for models (that is, MCSim) into a rapid, compilable (C) language. A public domain guide to MCSim is available at:* [*https://www.gnu.org/software/mcsim/mcsim.html*](https://www.gnu.org/software/mcsim/mcsim.html)

- Improve Table and Figure Presentation: Several tables are overly complex and could be simplified or broken down. Table 2, in particular, is very dense. Consider splitting it into smaller, more focused tables. Figures 3, 4, and 5 are helpful but could benefit from clearer labels and more concise captions.

We have thoroughly revised former table 2 (now table 3) so that it fits on a single page. We believe it is a critical part of the manuscript. It is intended to cross-walk between how aspects of a model are described in the three languages used by httk. We hesitate to break it into sub-tables because it will not serve its intended purpose if broken up.

We have increased the font sizes in Figure 3 and reduced the captions for Figures 3, 4, and 5.

- Reduce Redundancy: There is some redundancy in the manuscript, particularly regarding the descriptions of core functions and data tables. Streamline these discussions and avoid repeating information already presented in the tables.

Wherever we could, we have reduced duplicative material.

- Clarify the Intended Audience: Is this manuscript intended for experienced modelers, R users, or a broader audience? Clarify the intended audience and adjust the level of detail and explanation accordingly.

We have revised the concluding paragraph of the introduction to hopefully clarify this:

*We are inviting the biological modeling community to help expand the physiology and chemical domains addressed by httk. This work is intended to help the developers of new models use the existing HTTK data and functionality.*

## Minor Revisions:

- Carefully proofread the manuscript for typos and grammatical errors.

Thank you for the suggestions, multiple coauthors have checked for typos and grammar.

- Ensure consistency in terminology and abbreviations.

We have done our best.

- Provide more context and explanation for equations and mathematical concepts.

We have done our best.

- Double-check the accuracy of all URLs and references.

References and URLs have been checked.

- By addressing these major revisions, the manuscript can be significantly improved and provide a truly practical and accessible guide for integrating new TK models into httk, fostering community contributions and advancing the field of HTTK modeling

Thank you.

# Reviewer #3

- Please remove the quote from the end of the Introduction section. While Dr Feynman is the best, and I absolutely love the quote itself, it seems out of place and unnecessary here.

Done.

- A lot of the Methods section feels like it belongs in the Supplementary section

While we have extensively reorganized the Methods section and reduced duplicative material to address concerns from other reviewers, we are hesitant to move too much material to the Supplement. This is intended to be a “how to” paper and we hope to provide sufficient detail in the main manuscript.

# Reviewer #4

In their present work, Sarah et al. describe the integration and evaluation of new physiologically based toxicokinetic (PBTK) models into an open-source R package. The research is robust, the manuscript is well-structured, and the authors have meticulously detailed each step of the model development process. I would like to offer a few suggestions to further enhance the manuscript:

- Abstract: The abstract is quite lengthy. It would be beneficial to make it more concise while retaining the essential details.

We have rewritten and shortened the abstract.

- It would be helpful if the authors could discuss whether a similar tool is available in other programming languages, such as Python.

It is certainly possible to use MCSim with Python ([Lohitnavy](https://ieeexplore.ieee.org/author/37085348889) et al., 2017) and it is also possible to use R package httk through Python using RPy for Python (Kapraun et al., 2017). However, as far as we know the only open source database of chemical-specific HTTK parameters is R package httk. Python package ctx-python (<https://github.com/USEPA/ctx-python>) allows retrieval of pre-computed predictions made with httk from the CompTox Chemicals Dashboard. We have added this point to the Discussion:

*While there is no reason HTTK data and models must be implemented in R, we are unaware of comparable tools in other, similar languages such as Python. It is certainly possible to use MCSim with Python [79] and it is also possible to use R package httk through Python using rpy2 [80] for Python [81]. However, as far as we know the only open-source database of chemical-specific HTTK parameters and models is R package httk. Python package ctx-python (https://github.com/USEPA/ctx-python) allows retrieval of pre-computed predictions made with httk from the CompTox Chemicals Dashboard [63]. R package ctxR [82] provides functionality similar to ctx-python for R.*

- The resolution of Figure 2 is quite low. Replacing it with a higher-resolution image would significantly improve its clarity and visual appeal.

We have added a standalone copy of Figure 2 to the submission. The image is saved as a tiff file that we can provide to the journal at arbitrary resolution. We have doubled the resolution to 1200 x 800, but for the time being the version that builds in the PDF may show at lower than optimal resolution.
