# Supplementary material for: Enabling transparent toxicokinetic modeling for public health risk assessment
Source: PLoS One. 2025 Apr 16;20(4):e0321321. doi: 10.1371/journal.pone.0321321 (PMC12002443; doi:10.1371/journal.pone.0321321)
Supplement: Attachment — Submitted filename: Rebuttal Letter.docx [file pone.0321321.s011.docx]

Rebuttal Letter PONE-D-24-38287

Enabling Transparent Toxicokinetic Modeling for Public Health Risk Assessment

*A rebuttal letter responds to each point raised by the academic editor and reviewer(s).*

# Associate Editor

Thank you for considering our journal for publication of your manuscript. Your manuscript has now been peer-reviewed, could you please take their comments into account and submit a revised version of your manuscript for our consideration?

Please submit your revised manuscript by Feb 06 2025 11:59PM.

# Reviewer #1

This manuscript describes the integration and evaluation of new PBTK models into an existing R package (httk) in order to improve in vitro to in vivo extrapolation, which is critical for human health risk assessment. The manuscript is written exceptionally well with clear referals to case studies and model performance evaluations. The manuscript also addresses limitations of the existing package and the improvements that will be implemented by the authors. I do not have any major queries that would impact on the current standard of the manuscript and would therefore suggest acceptance of the manuscript without revision.

# Reviewer #2

This manuscript describes the process of adding new TK models to the httk R package. The topic is relevant, particularly given the increasing reliance on in vitro and in silico methods for chemical risk assessment.

- While the manuscript provides a valuable overview of httk and its functionalities, several major revisions are needed to enhance clarity, accessibility, and impact. Specifically, the manuscript needs to be more concise and focused on the practical aspects of model integration, with less emphasis on background information already available elsewhere.
- A more concrete and worked-through example is essential for readers to grasp the process.

## Major Revisions

- Streamline the Introduction: The introduction is lengthy and contains substantial background on HTTK and risk assessment that could be condensed or removed. Focus on the specific gap this manuscript addresses – the need for clear guidelines on integrating new models into httk – and the benefits of doing so (e.g., community contributions, model standardization, improved reproducibility).
- Simplify and Refocus the Results Section: The current results section blends model integration details with evaluation results. Separate these clearly. The re-evaluation of the Linakis et al. model is interesting but distracts from the core purpose of the manuscript. Either move this to a supplementary document or significantly shorten it, focusing on specific changes and their impact on RMSLE. Emphasize the practical implications of observed changes for model developers. The discussion of benchmark_httk() is more appropriate for the methods.
- Provide a Concrete, Worked-Through Example: The most significant weakness is the lack of a clear, step-by-step example. While the gas inhalation model is mentioned and supplementary files are provided, the manuscript doesn't guide the reader through the process of integration. Include a simplified, illustrative example within the main text, demonstrating the creation of a basic model (even a one-compartment model) and its integration into httk. Show concrete code snippets and explain the purpose of each step. This example should be the central focus of the manuscript.
- Reorganize the Methods Section: The methods section is currently quite dense and difficult to follow. Reorganize it into more logical subsections with clear headings, following the steps of model integration described in Table 1. Each subsection should describe the specific tasks involved in that step, the relevant httk functions and data, and provide short, illustrative code examples where appropriate. Move the discussion of benchmark_httk() from the Results to the "Model Evaluation" subsection of the Methods.
- Clarify the Role of MCSim: The manuscript mentions using MCSim for model description but doesn't clearly explain its advantages or provide enough guidance on its use. Expand this discussion and provide a simple MCSim example within the worked-through example mentioned above. If the authors intend to encourage MCSim use for new httk model contributions, this warrants more attention.
- Improve Table and Figure Presentation: Several tables are overly complex and could be simplified or broken down. Table 2, in particular, is very dense. Consider splitting it into smaller, more focused tables. Figures 3, 4, and 5 are helpful but could benefit from clearer labels and more concise captions.
- Reduce Redundancy: There is some redundancy in the manuscript, particularly regarding the descriptions of core functions and data tables. Streamline these discussions and avoid repeating information already presented in the tables.
- Clarify the Intended Audience: Is this manuscript intended for experienced modelers, R users, or a broader audience? Clarify the intended audience and adjust the level of detail and explanation accordingly.

## Minor Revisions:

- Carefully proofread the manuscript for typos and grammatical errors.
- Ensure consistency in terminology and abbreviations.
- Provide more context and explanation for equations and mathematical concepts.
- Double-check the accuracy of all URLs and references.
- By addressing these major revisions, the manuscript can be significantly improved and provide a truly practical and accessible guide for integrating new TK models into httk, fostering community contributions and advancing the field of HTTK modeling

# Reviewer #3

- Please remove the quote from the end of the Introduction section. While Dr Feynman is the best, and I absolutely love the quote itself, it seems out of place and unnecessary here.

Done.

- A lot of the Methods section feels like it belongs in the Supplementary section

# Reviewer #4

In their present work, Sarah et al. describe the integration and evaluation of new physiologically based toxicokinetic (PBTK) models into an open-source R package. The research is robust, the manuscript is well-structured, and the authors have meticulously detailed each step of the model development process. I would like to offer a few suggestions to further enhance the manuscript:

- Abstract: The abstract is quite lengthy. It would be beneficial to make it more concise while retaining the essential details.
- It would be helpful if the authors could discuss whether a similar tool is available in other programming languages, such as Python.
- The resolution of Figure 2 is quite low. Replacing it with a higher-resolution image would significantly improve its clarity and visual appeal.
